# Comparing Colon Capsule Endoscopy to colonoscopy; a symptomatic patient’s perspective

**DOI:** 10.1186/s12876-021-02081-0

**Published:** 2022-01-24

**Authors:** Mohd Syafiq Ismail, Greg Murphy, S. Semenov, D. McNamara

**Affiliations:** 1 Department of Gastroenterology, Tallaght University Hospital, Dublin, Ireland; 2 Trinity Academic Gastroenterology Group (TAGG), The Trinity Centre for Health Sciences, Tallaght University Hospital & Trinity College Dublin, Dublin 24, Ireland

**Keywords:** Colon Capsule Endoscopy, Colonoscopy, Patient preference, Comfort

## Abstract

**Background:**

Colon Capsule Endoscopy (CCE) has proven efficacy in a variety of gastrointestinal diseases. Few studies have assessed patient-reported outcomes and preference between colonoscopy and CCE.

**Methods:**

Patients from our centre who had both a CCE and colonoscopy within a 12-month period were identified. We performed over-the-phone interviews focused on satisfaction, comfort, and overall preference with a 10-point Likert scale. Electronic records were reviewed; reported Modified-Gloucester-Comfort-Scale (GCS) score, sedation, bowel preparation and endoscopist grade were documented. Data was compared between procedures. A Fishers exact test was used to compare proportions and a Student t-test was used to compare means, a *p* < 0.05 was considered significant.

**Results:**

In all, 40 patients were identified, 57.5% (23/40) were female and the mean age was 48 years (24–78). All patients were referred for investigation of lower gastrointestinal symptoms as part of an ongoing study [Endosc Int Open. 2021;09(06):E965–70]. There was a significance difference in mean comfort (9.2 vs 6.7, *p* < 0.0001, 95% CI − 3.51 to − 1.44) but not satisfaction (8.3 vs 7.7, *p* = 0.2, 95% CI − 1.48 to 0.33) between CCE and colonoscopy. Main cause of dissatisfaction with CCE was bowel preparation and for colonoscopy was discomfort. Age and gender were not found to be variables. The correlation between GCS and patient reported values was weak (R = − 0.28). Overall, 77.5% (31/40) of patients would prefer a CCE if they required further bowel investigation. Of these, 77.4% (24/31) preferred a CCE despite the potential need for follow-up colonoscopy.

**Conclusions:**

CCE has a high satisfaction rating (8.3 vs 7.7) and has a higher patient reported comfort rating (9.2 vs 6.7) than colonoscopy. Studies have confirmed CCE and colonoscopy have equivalent diagnostic yields. The majority of patients in our cohort prefer CCE to colonoscopy. CCE should be considered as an alternative to colonoscopy in selected individuals.

## Background

Lower gastrointestinal symptoms such as chronic diarrhoea and bleeding per rectum (PR) are a common cause for referral to the gastroenterology department. As symptoms alone are poor at predicting clinically significant disease, symptomatic patients are often referred for further investigations which may include a colonoscopy [1]. Fibreoptic colonoscopy is a well-established modality for investigating the large bowel and offers both diagnostic and therapeutic potential for ileocolonic diseases.

Colonoscopies are performed in dedicated endoscopy units. In Ireland, they are usually performed under conscious sedation with Midazolam and Fentanyl. Even though colonoscopies are generally well tolerated and considered safe, they do come with potential risks. General complications associated with colonoscopies are usually related to sedation, bowel perforation and a risk of haemorrhage. A recent review article by the American Society of Gastrointestinal Endoscopy (ASGE) found a pooled perforation rate of 5.8 per 10,000 colonoscopies (95% CI 5.7–6.0), bleeding rate of 2.4 per 1000 colonoscopies (95% CI 2.4–2.5), and death rate of 3 in 100,000 colonoscopies [2]. Furthermore, there is a risk of causing pain and discomfort to the patient during colonoscopy. This is often worse in younger, female patients with lower Body Mass Indices (BMI) or in patients who have had previous abdominal and pelvic surgery [3].

Apart from the risks of complications, there are also patient factors to be considered. Patients can be reluctant to undergo this invasive procedure due to their perceived embarrassment, inconvenience, or discomfort associated with colonoscopy [4, 5]. In a study about anxiety related to colonoscopy in 1336 patients, Shafer et al. found very high anxiety scores (> 70) reported by 29% of patients relating to the procedure itself [6].

The European Society of Gastrointestinal Endoscopy (ESGE) has recommended Colon Capsule Endoscopy (CCE) as a safe alternative to colonoscopy in average risk individuals [7]. In a more recent joint guideline update, by ESGE and the European Society of Gastrointestinal and Abdominal Radiology (ESGAR) CCE may be considered as an alternative to colonoscopy in patients with non-alarm symptoms [8]. CCE is considered less invasive and does not require air insufflation or sedation. Risks of CCE are minimum and has recently been reported in a meta-analysis by Wang et al. as capsule retention (0.26%), swallowing difficulties (0.04%), technical failure (1.76%), incomplete colonic examination (19%), and discomfort (0.81%) [9].

CCE has proven efficacy to detect both neoplastic and non-neoplastic disease in selected patient cohorts including Inflammatory Bowel Disease patient assessment, average and low risk colorectal cancer screening, after incomplete colonoscopy and polyp surveillance [10–18]. A recently published study from our centre also has shown that CCE had a sensitivity, specificity, positive predictive value (PPV), negative predictive value (NPV) of 81%, 98%, 93% and 94% respectively in a symptomatic cohort when compared to colonoscopy for detecting clinically significant disease [19]. Despite its growing popularity and proven clinical efficacy, few studies have focused on patient preference between colonoscopy and CCE.

### Aims

The aim of this study was to identify patient reported outcomes of comfort and satisfaction in relation to both CCE and colonoscopy and to establish if there was a difference in preference between both modalities. We also wanted to assess whether there was a difference in patient versus endoscopist reported comfort scores post colonoscopy.

## Method

This study has been done in accordance with STROBE (STrengthening the Reporting of OBservational studies in Epidemiology) guidelines. This is a retrospective single centre comparative study conducted in Tallaght University Hospital (TUH), Ireland of patient reported satisfaction and comfort for CCE and colonoscopy procedures. TUH is an established CCE centre in Ireland, we perform about 250–300 CCEs per year in accordance with ESGE guidance and for ethically approved research, including in this symptomatic cohort.

This study has been approved as a service assessment by the quality improvement committee, Tallaght University Hospital.

### Study Population and referral criteria

Identified patients between the age of 18–80 from our electronic database who had both a CCE and colonoscopy over a 12-month period (Dec 2017–Dec 2018) was included in this study. All patients have given verbal consent to participate in this study. In order to prevent selection bias, all contactable patients who had both tests over the period were recruited. We excluded patients who could not consent to the study.

Both CCE and colonoscopy tests were performed as part of another prospective comparison study of CCE and colonoscopy for the investigation of symptoms in an average risk cohort [19]. As such all participants were scheduled to have both procedures. More detailed description of this cohort has already been published.

### Study Design

A summary of the study is outlined in Table 1. All CCEs were performed using PillCam™ COLON 2 (Medtronic, Minneapolis, USA). Colonoscopy reports were generated and stored on a national endoscopy database, Unisoft (HD Clinical, Hertfordshire, UK).

**Table 1:** Study design and patient demographics

Study design
**Step 1: Patient identification through electronic database from Dec 2017 to Dec 2018**
N = 40 patients identified
57.5% (23/40) female,
Mean age—48 (24–78) years
**Step 2: Patient contacted for interview (Appendix A)**
**Step 3: Further data collection for CCE and colonoscopy**
CCE data—Bowel preparation and booster medications, findings, capsule completion rate
Colonoscopy data—GCS from colonoscopy, endoscopist grade, sedation used, findings, completion, other adjuncts used

Upon recruitment, we performed over-the-phone interviews a minimum of 3 months after completion of their investigations, focusing on questions related to satisfaction, comfort, and overall preference. Interviews were performed by trained medical professionals using secure hospital phone lines. Interview questions are listed in Appendix A. A 10-point Likert scale was used to assess both comfort and satisfaction ranging from 1 (negative response) to 10 (positive response) for both CCE and colonoscopy procedures. The interviewers also asked each interviewee, if further colonic investigations were required, which test if either they would prefer; a CCE or colonoscopy. If patients had answered CCE as the preferred method of investigation, based on their own personal experience, the interviewer then asked if their answer would change if the CCE found findings that warranted referral on for a colonoscopy including incomplete CCE. The interviewer then asked an open-ended question regarding any cause of dissatisfaction for either CCE or colonoscopy.

In addition to the interview, we collected further demographic, clinical and procedural data for each patient from our CCE and endoscopy databases including bowel preparation and booster regimens, and capsule completion rate. Further colonoscopy data collected included, the Modified-Gloucester-Comfort-Scale (GCS) score, endoscopists grade and quality data (caecal intubation rate—CIR, polyp detection rate—PDR, and mean comfort score ≤ 3), amount of sedation used, and whether a scope guide was used during colonoscopy.

The GCS is used at our centre and in all endoscopy units in Ireland. This score is usually given by the endoscopy nurses after the colonoscopy and agreed by the endoscopist in order to prevent an operator bias. As detailed in Appendix B, a score of 1 refers to no discomfort while 5 is associated with severe discomfort. These scores are kept on a secure database and form part of the quality indicators for colonoscopy which is regularly audited as part of the National Quality Assurance Information System (NQAIS). This data is not available on endoscopy reports and is not routinely told to the patients post procedure. There was no endoscopist reported comfort score for CCE.

### Bowel preparation

Bowel preparation for CCE was a split dose 4-L (4L) Polyethylene glycol (PEG) (KleanPrep—Norgine, Middlesex, UK) with the addition of Phospho-soda, “Fleet” (Casen Recordati, Zaragoza, Spain), Gastrograffin (Bayer, Reading, UK) and rectal Bisacodyl (Dulcolax—Sanofi-Aventis, Ireland) suppository booster medications. Similarly, all patients received a further split dose 4L Polyethylene glycol (KleanPrep—Norgine, Middlesex, UK), bowel preparation prior to colonoscopy (Appendix C). In addition, standard dietary manipulation was advised for several days prior to both procedures.

All obtained data were kept on secure encrypted hospital computers. Statistical analysis was performed using MedCalc (MedCalc Software Ltd, Belgium). A Chi-squared test was used to compare categorical data and a Student t-test was used to compare means, a *p* < 0.05 was deemed significant. Pearson Correlation Coefficient was used to assess the correlation between patient and operator reported comfort scores. A r value of ≥ 0.7 was considered as a strong correlation.

## Results

### Study Population

In all, 40 patients were identified at our centre who had both a CCE and colonoscopy over the 12-months period. All the patients were contacted and were willing to participate in the over-the-phone interviews; 57.5% (23/40) of these were female, with a mean age of 48 (24–78) years. All CCE tests, were performed before the colonoscopy. The interval between the two procedures was on average 6 weeks (range 2–8 weeks). The CCE report was available to the endoscopist at the time of colonoscopy.

### CCE data

CCE completion rate was 78% (n = 31) with 100% (n = 40) reaching the left colon. Positive findings were reported in 68% (n = 27). Bowel preparation quality for CCE were reported as good/adequate in 36 (90%) and inadequate in 4 (10%).

### Colonoscopy data

All colonoscopies were performed by two senior endoscopists who met NQAIS standards with CIR of 95.5%, 95.3%, PDR of 42.4%, 49.9% and mean GCS ≤ 3 of 99%, 96% respectively. All patients received procedural sedation with mean sedation of 4.2 mg (range 2–8 mg) of Midazolam and 68.1mcg (range 50–100 mcg) of Fentanyl. Caecal intubation was achieved in 92.5% (n = 37). The scope guide was not used in any of the colonoscopy procedures. All colonoscopies were done under air insufflation only. Positive findings were reported in 65% (n = 26). Bowel preparation quality for colonoscopy was reported as good/adequate in 36 (90%) and inadequate in 4 (10%). Total colonoscopy procedural times were not available.

There were no immediate or late procedural related complications for any patients for both their CCE and colonoscopy procedures. All patients were reviewed in clinic following their tests and appropriate follow up arranged.

### Interview results

The mean interval between CCE to interview was 33 weeks (14 to 52 weeks) and the mean interval between colonoscopy to the interview was 30 weeks (12 to 50 weeks).

Regarding satisfaction score, mean satisfaction reported was 8.3 (range 3–10) for CCE and 7.7 (range 1–10) for colonoscopy. Regarding comfort score, mean comfort was 9.2 (range 6–10) for CCE vs 6.7 (1–10) for colonoscopy.

There was a statistically significant difference in mean comfort (9.2 vs 6.7, *p* < 0.0001, 95% CI − 3.51 to − 1.44) but not satisfaction scores (8.3 vs 7.7, *p* = 0.2, 95% CI − 1.48 to 0.33) between CCE and colonoscopy respectively.

Of note the volume of bowel preparation and booster medications required was identified as the main cause of dissatisfaction with CCE (25%, n = 10/40). The main cause of dissatisfaction with colonoscopy was pain and discomfort (33%, n = 13/40).

### Subgroup analysis

There was no significant differences between mean satisfaction for either CCE or colonoscopy when responses were divided into gender (male and female) or age (age less than 50 and above 50).

However, when looking at comfort, there were significant differences between responses in all subgroups favouring CCE over colonoscopy. The highest significant difference was between the comfort reported in those below 50 favouring CCE over colonoscopy (9.4 vs 6.5, *p* =  = 0.0002, 95% CI − 4.21 to − 1.55).

A further breakdown of subgroup analysis is listed in Table 2.

**Table 2:** Mean difference between patient reported scores for CCE and Colonoscopy

	CCE	Colonoscopy	*P* value	95% CI
*Satisfaction*	8.3	7.7	*p* = 0.20,	− 1.48 to 0.33
Male	8	8	*p* = 1,	− 1.06 to 1.06
Female	8.4	7.4	*p* = 0.16	− 2.41 to 0.41
< 50	7.8	7.9	*p* = 0.80	− 0.83 to 1.07
> 50	9	7.3	*p* = 0.06	− 3.56 to 0.1
*Comfort*	9.2	6.7	*p* < 0.0001	− 3.51 to − 1.44
Male	9.2	6.9	*p* = 0.004	− 3.75 to − 0.84
Female	9.2	6.6	*p* = 0.002	− 4.16 to − 1.06
< 50	9.4	6.5	*P* = 0.0002	− 4.21 to − 1.55
> 50	8.9	7.1	*p* = 0.05	− 3.62 to 0.02

### Patient preference

Overall, 77.5% (n = 31/40) of patients would prefer CCE if they required a further lower GI investigation in the future. Of these, 77.4% (n = 24/31) still preferred CCE despite the potential need for a follow-up colonoscopy. 70.6% (12/17) of males and 82.6% of females prefer CCE over colonoscopy and gender does not seem to affect this preference (*p* = 0.37).

The mean reported Modified-Gloucester-Comfort-Scale for colonoscopies were 1.4 (range 1–4). When looking at the correlation between endoscopist reported GCS mean 1.4 (range 1–4) and patient reported scores 6.7 (1–10), only a weak negative correlation was found (r = − 0.28). If we assume that comfort was defined as a GCS < 3, and a 10-point scale score ≥ 7, 73% (n = 29) of patients reported feeling comfortable with their colonoscopy while endoscopy reported comfort was 88% (n = 35) with a weak positive correlation reported (r = 0.44, *p* = 0.004).

## Discussion

Our retrospective comparative study has found that CCE was reported by patients to be a more comfortable procedure (9.2 vs 6.7, *p* < 0.0001) with comparable satisfaction scores compared to colonoscopy (8.3 vs 7.7, *p* = 0.28). A majority of our patients (77.5%, n = 31/40) also preferred to have a CCE compared to colonoscopy if a repeat endoscopic procedure is required in the future. Among these, a further 77.4% (24/31) of patients still preferred a CCE over colonoscopy even if the results of the CCE would require them to have a colonoscopy for any intervention or further diagnostic work-up.

Both procedures resulted in similar satisfaction scores (CCE − 8.3 vs colonoscopy − 7.7, *p* = 0.28). For CCE, high satisfaction score could be related to the minimally invasive nature of the procedure, the fact that no/minimal discomfort or pain is involved, and the lack of embarrassment associated with colonoscopy. Furthermore, in our centre, CCEs are performed in our dedicated Capsule Endoscopy Department by trained nursing staff, medical staff, and technicians as outpatient procedures. As patients are not given any sedative medications for CCE, they can go about their day as normal, with some patients opting to return to work while others seek the comfort of their own homes. For colonoscopy, the high satisfaction score could be related to the professionalism of the endoscopy department staffs, the definitive nature of colonoscopy in most cases, and the ability to perform intervention on same procedure. The absence of complications in our cohort also could be associated with high satisfaction scores.

Of note for both procedures, patients are usually contacted prior to attending the procedure to ensure compliance with bowel preparation and diet, and to address any concerns that they may have, which could have contributed to similarly high overall satisfaction.

Despite having higher comfort and satisfaction scores, the amount of bowel preparation and booster medications (Appendix C) that needed to be taken was identified as the main issue causing dissatisfaction with CCE (25% of patients). CCE relies on a high degree of bowel cleansing as it lacks the air insufflation and the ability to perform colonic cleaning and suctioning in colonoscopy. Apart from that, unlike for a Small Bowel Capsule Endoscopy (SBCE), booster medications are needed to propel the capsule through a patient’s small bowel and colon to ensure a complete examination. This regimen, although appearing excessive, is based on the 2012 ESGE guidelines for CCE [7]. Since the completion of this study, CCE preparation in our unit has been changed to a lower volume split dose MoviPrep (Norgine, Middlesex, UK) preparation pre-CCE, with MoviPrep and Castor oil as boosters. Audits of these changes have shown an 87% capsule excretion rate and also improved image quality [20, 21]. We have yet to assess patient’s satisfaction and comfort following this change but due to reduction of the volume of bowel preparation, one would assume higher scores will be awarded.

This study also highlights the disconnect between the level of comfort patients experienced compared to endoscopy reported Modified-Gloucester-Comfort-Scores. The GCS, although not formally validated, is used to assess patient comfort during colonoscopy in all Irish endoscopy units, forms part of quality indicators of colonoscopy and is regularly audited by NQAIS [22–24]. Similar discordances have also been reported in other studies [25, 26]. The GCS was designed to be provided by the endoscopy nurses and agreed by the endoscopist, but it is possible that they are too intimately involved in the procedure to provide a truly objective assessment [25]. Poor comfort outcomes may cause patients to refrain from engaging with similar diagnostic tools in the future. Development and implementation of comfort scores that includes patient’s perspective such as the Patient-Reported Scale for Tolerability of Endoscopic Procedures (PRO-STEP) [27] or scores that have shown correlation with patient reported outcomes such as the St. Paul's endoscopy comfort score (SPECS) [22] should be considered. Although these scores are validated their use are not yet common. GCS itself has never been validated for patient use. To date, there isn’t a validated scoring system that measures both patients reported satisfaction and comfort. This is why we chose the simpler 10-point Likert scale in our interview for better patient comprehension and understanding.

The strength of this study is that to our knowledge, it is the only study that purely focuses on patient reported outcomes following both CCE and colonoscopy procedures in a symptomatic cohort. In a mix of screening and symptomatic patients, Ojidu et al. reported in different group of patients undergoing either CCE, colonoscopy and CT colonography (CTC) mean GCS’s of 1.3, 3.32, and 1.96 respectively [25]. Furthermore, when comparing CCE and CTC, they also found that 76.8% reported no discomfort for CCE compared to 31.9% for CTC [25]. In a CRC screening cohort, Thygesen et al. reported moderate to high discomfort in 89% participants undergoing colonoscopy compared with7% undergoing CCE [28]. It is interesting to note that despite receiving intravenous procedural sedation and analgesia for colonoscopy patient reported comfort scores are poorer, reflecting the invasive nature of this procedure.

Patient reported outcome studies may be prone to certain individual biases, however it is the only way to assess patients own perspective regarding satisfaction and comfort.

We did not report on any patient diagnosis in this study and purely focused on patient reported procedural outcomes of comfort and satisfaction. The impact of diagnosis on the overall satisfaction and perceived comfort is not known. However, it should not significantly affect the results as patients were unselected and average risk and there were no cases of cancer detected.

An advantage is that all our participants were in a comparative study and underwent both tests giving subjects the opportunity to compare both procedures directly. This could however be considered not to reflect their preferences in actual clinical practice. In particular, the need for a therapeutic colonoscopy after a positive CCE could be a disadvantage. In our cohort though, the majority who reported a preference for CCE in the future, did so accepting of that possibility. In addition, this argument supposes there is never a need for a repeat colonoscopy, for preparation quality, anticoagulant or completion issues. As such the opportunity to directly compare tests remains a valid assessment.

Another possible limitation to this study is that interviews were conducted at most 3 months since completion of both tests. The effect of this time lag on patients recall cannot be assessed. Another factor that may not be able to assess is the effect of sedation on patient comfort and recall in our study cohort. Ball et al. has previously reported that the amount of sedation given to a patient during colonoscopy does not correlate with comfort scores (ρ < 0.2) [29].

In terms of other factors that may be associated with comfort scores, it has been previously reported that symptomatic patients often experience poorer comfort scores than screening patients [25], whether or not this played a role in our cohort is difficult to determine. The use of Carbon Dioxide (CO2) insufflation has been associated with better comfort scores [30]. We only used air insufflation and not CO2 insufflation in our colonoscopies during this study period due to unavailability and hence would be a limitation to this study. Since study completion, all endoscopy rooms in our department have been installed with a CO2 insufflation device. The correlation of its use and comfort score in our centre has yet to be reviewed. Both of our endoscopists performing the colonoscopies met and exceeded minimum NQAIS standards. The use of the scope guide has not been found to be beneficial in improving patients comfort scores in experienced endoscopists and is unlikely to have affected our results [31].

In our cohort, we did not find a difference between patients reported satisfaction and comfort when looking at age (less than and above 50) and gender as possible variables. We did not have available data to assess patients’ anxiety levels pre-procedure, duration of colonoscopy, patients BMI and endoscopist reported ease of colonoscopy, all of which may be possible factors that may result in a more difficult and uncomfortable procedure [3]. This does open possibility for future prospective research.

As mentioned previously, 77.5% (31/40) of patients would prefer to have CCE over colonoscopy in the future. Interestingly of these 31 patients, 77.4% (24/31) would still choose to have CCE as first line even with the knowledge that they may have to have a colonoscopy as a second procedure if further investigation or intervention was required. As explained in the introduction, CCE has proven efficacy in a variety of clinical situations and can be used in both the symptomatic and screening cohort [10–18]. As such, we would like to recommend clinicians include alternatives to colonoscopy such as CCE when discussing available investigations with patients. Providing proven alternatives, especially ones that are associated with greater levels of comfort and less complications should form part of the process of informed consent.

## Conclusions

CCE has a high satisfaction rating and has a higher comfort rating than colonoscopy in symptomatic patients. Studies have confirmed CCE and colonoscopy have equivalent diagnostic yields. The majority of patients in our cohort prefer CCE to colonoscopy. CCE should be considered as an alternative to colonoscopy in selected individuals.

## Data Availability

The datasets used and/or analysed during the current study are available from the corresponding author on reasonable request.

## Appendix A: Patient Questionnaire

**Table:**

Patient Questionnaire
*On a scale of 1–10 (1 = Negative / 10 = Positive)*
How satisfied were you with your CCE?
How satisfied were you with your colonoscopy?
In terms of pain, how comfortable was your CCE?
In terms of pain, how comfortable was your colonoscopy?
*Preference*
If you required further bowel testing in the future, would you rather a CCE or Colonoscopy?
*Follow on Question (if above = “CCE”)*
Would your answer change if there was a likelihood/chance that you would require a colonoscopy following CCE (i.e. have both tests)?
Any particular reasons for your dissatisfaction with CCE and/or colonoscopy?

## Appendix B: Modified-gloucester-comfort-scale

**Table:**

Score	Scale	Description
1	No	No discomfort, talking/resting comfortably throughout
2	Minimal	One or two episodes of mild discomfort (without distress)
3	Mild	More than two episodes of mild discomfort (without distress)
4	Moderate	Significant discomfort experienced several times with some distress
5	Severe	Extreme discomfort frequently during the test

## Appendix C: Bowel preparation regimen for CCE and colonoscopy

**Table:**

Procedure	Bowel preparation	
CCE	1 week before test	Stop all iron tablets
	Day − 2 (Day minus two, 2 days prior to study)	4 × 7.5 mg Senna tablets at 7 pm
	Day − 1 (Day minus one, 1 day prior to study)	Liquid diet throughout the day (includes black tea, black coffee, broth (no particles) etc
		Drink at least 10 glasses of water
		2 L of KleanPrep (PEG) 7 pm
		Fast from midnight
	Day 0 (test Day): 6am	2 L of KleanPrep (PEG)
	9am	CCE is swallowed
	Small bowel reached (normally takes approx. 20–30 min, patient is encouraged to walk around, if delayed in the stomach 10 mg of IV Metoclopramide can be given, failing that options include endoscopic placement)	1st booster of Sodium Phosphate (Fleet) 45mls (mix sachet with standard 150mls of water and extract 45mls)
		+ 50mls of Gastrograffin mixed with 1 L of water
		Patient can leave the department. Instructions for 2nd booster should be explained prior to departure
	3 h later	2nd booster of Sodium Phosphate (Fleet) 25mls (prepare as per booster 1)
	2nd bowel booster prepared by patient	+ 25mls of premeasured Gastrograffin and 500mls of water
		Patient can eat and drink as normal at this point
	2 h post 2nd booster	If capsule not passed, rectal bisacodyl suppository used
Colonoscopy	1 week before test	Stop all iron tablets
	Day − 1 (Day minus one, 1 day prior to study)	Liquid diet throughout the day (includes black tea, black coffee, broth (no particles) etc.)
		Drink at least 10 glasses of water
		2 L of KleanPrep (PEG) 7 pm
		Fast from midnight
	Day 0 (test Day): 6am	2 L of KleanPrep (PEG)

## Abbreviations

TAGG: Trinity Academic Gastroenterology Group
CCE: Colon Capsule Endoscopy
GCS: Modified-Gloucester-Comfort-Scale
PR: Per rectum
ASGE: American Society of Gastrointestinal Endoscopy
ESGE: European Society of Gastrointestinal Endoscopy
ESGAR: European Society of Gastrointestinal and Abdominal Radiology
PPV: Positive predictive value
NPV: Negative predictive value
STROBE: STrengthening the Reporting of OBservational studies in Epidemiology
TUH: Tallaght University Hospital
CIR: Caecal intubation rate
PDR: Polyp detection rate
NQAIS: National Quality Assurance Information System
PEG: Polyethylene glycol
SBCE: Small Bowel Capsule Endoscopy
PRO-STEP: Patient-Reported Scale for Tolerability of Endoscopic Procedures
SPECS: St. Paul's endoscopy comfort score
CTC: CT colonography
CO2: Carbon dioxide
